# High-Capacityand Reversible Hydrogen Storage in an Intrinsic Li_3_B_2_N_2_ Monolayer

**DOI:** 10.3390/nano16110654

**Published:** 2026-05-23

**Authors:** Haichuan Yu, Jingyan Chen, Jian Hao, Caoping Niu, Meiling Xu, Yinwei Li

**Affiliations:** Jiangsu Key Laboratory of Extreme Multi-Field Materials Physics, School of Physics and Electronic Engineering, Jiangsu Normal University, Xuzhou 221116, China; 3020244609@jsnu.edu.cn (H.Y.); 2020231335@jsnu.edu.cn (J.C.); yinwei_li@jsnu.edu.cn (Y.L.)

**Keywords:** first-principles, hydrogen storage, adsorption energy, storage capacity, desorption temperature, metal-decoration-free

## Abstract

Hydrogen is widely considered a promising clean energy carrier because of its high energy density and environmental benignity, yet the development of safe and reversible hydrogen storage materials remains a major challenge. Two-dimensional materials are particularly attractive for this purpose owing to their large specific surface area, fully exposed active sites, and highly tunable electronic structures. Here, using crystal structure prediction combined with first-principles calculations, we predict a stable metallic Li_3_B_2_N_2_ monolayer as a potential hydrogen storage material. This monolayer can adsorb up to six H_2_ molecules per unit cell with an average adsorption energy of ∼0.23 eV/H_2_, yielding a high hydrogen storage capacity of ∼7.8 wt.%. Further analysis reveals that hydrogen adsorption is governed by the synergistic effects of electrostatic polarization and orbital hybridization. Moreover, calculations on the temperature- and pressure-dependent hydrogen storage behavior show that all hydrogen-adsorbed structures remain stable at room temperature under a pressure of 3.7 MPa. The van’t Hoff analysis indicates that the maximum desorption temperature at atmospheric pressure is 316 K, suggesting favorable reversibility under near-ambient conditions. These results establish Li_3_B_2_N_2_ as a promising intrinsic two-dimensional material for high-density and reversible hydrogen storage.

## 1. Introduction

As fossil fuel resources continue to be depleted and environmental concerns intensify [1,2], hydrogen has emerged as a promising clean energy carrier owing to its abundance, high energy density, and zero-carbon emissions [3]. However, the efficient storage and safe transportation of hydrogen remain critical challenges for the realization of a hydrogen economy. Traditional hydrogen storage methods, such as high-pressure gaseous storage [4] and cryogenic liquid storage [5], require stringent safety measures and are often accompanied by high energy consumption and serious boil-off losses, which greatly hinder the large-scale development and application of hydrogen energy. In this context, solid-state hydrogen storage, which relies on the physical or chemical adsorption of hydrogen within materials, offers distinct advantages in terms of safety, volumetric density, and reversibility, and is therefore regarded as a promising strategy for future hydrogen storage.

For practical applications, solid-state hydrogen storage materials are expected to satisfy the U.S. Department of Energy (DOE) target of 5.5 wt.% for hydrogen storage capacity [6], together with the optimal adsorption energy of 0.2–0.6 eV/H_2_ proposed by Kim et al. [7], so as to enable reversible hydrogen adsorption and desorption under near-ambient conditions. The emergence of two-dimensional (2D) materials, such as graphene [8], hexagonal boron nitride (*h*-BN) [9], MoS_2_ [10], and MXenes [11,12,13], has stimulated extensive research interest because of their large specific surface areas, excellent chemical stability, and low weight, all of which are favorable for hydrogen storage. However, pristine 2D materials generally exhibit only weak physisorption toward hydrogen molecules, leading to limited storage capacities and poor reversibility under ambient conditions [14,15].

To improve hydrogen storage performance, external metal decoration has been widely adopted as an effective strategy because decorated metal atoms can generate an internal electric field that enhances the interaction with H_2_ molecules [16,17,18,19,20,21]. Among various metal species, lithium is particularly attractive because its low atomic mass is beneficial for achieving high gravimetric hydrogen storage capacity. For instance, Yong et al. [22] reported that Li decoration increases the hydrogen storage capacity of graphene from 0.4 wt.% to 12.8 wt.%, far exceeding the DOE target [6], which attributed to Li-induced polarization-enhanced H_2_ adsorption. Previous theoretical studies have also shown that metal-functionalized 2D materials, including Li@Irida-graphene [23], Li@γ-graphdiyne [24], Sc@2D polyaramid [25], Ti/Zr@2D polyaramid [26], and Li-functionalized $B_{x}$$C_{y}$$N_{z}$ compound [27,28,29,30,31,32,33], possess desirable adsorption energies (0.18–0.35 eV), high hydrogen storage capacities (7.06–14.66 wt.%), and favorable desorption behavior near room temperature. In addition, the hydrogen storage performance of the MXene Hf_2_CF_2_ [34] can be significantly enhanced by Li decoration, yielding a capacity of 10.8 wt.% through a charge-transfer-induced polarization mechanism.

Despite these advantages, externally decorated metal atoms often suffer from aggregation or clustering on 2D substrates, which can severely deteriorate the reversibility and cycling stability of hydrogen storage [35,36,37,38,39,40]. Therefore, identifying intrinsically high-performance 2D hydrogen storage materials that contain built-in active adsorption sites, rather than relying on post-synthetic metal decoration, remains an active and important topic in this field [41].

In this work, we predict a thermodynamically stable Li_3_B_2_N_2_ monolayer by combining crystal structure prediction with first-principles calculations. Unlike conventional metal-decorated 2D systems, the Li atoms in Li_3_B_2_N_2_ are intrinsic structural components, which provide built-in Li-rich adsorption sites for H_2_ molecules. The adsorption energies of H_2_ molecules on Li_3_B_2_N_2_ range from 0.20 to 0.37 eV/H_2_, well within the optimal energy window for reversible hydrogen storage. The Li_3_B_2_N_2_ monolayer can accommodate up to 18 H_2_ molecules in a 1×3×1 supercell, corresponding to a hydrogen storage capacity of 7.8 wt.%, which exceeds the DOE target. Based on the van’t Hoff equation, the average dehydrogenation temperature of Li_3_B_2_N_2_ is estimated to be 289 K at atmospheric pressure. These appealing properties establish the Li_3_B_2_N_2_ monolayer as a promising candidate for reversible hydrogen storage applications.

## 2. Computational Methods

The global structure prediction was performed using the Crystal structure AnaLYsis by Particle Swarm Optimization (CALYPSO) code [42,43,44,45]. Structural and electronic properties were calculated within the framework of density functional theory (DFT) using the Vienna Ab initio Simulation Package (VASP) [46,47]. The exchange-correlation functional was treated within the generalized gradient approximation (GGA) using the Perdew–Burke–Ernzerhof (PBE) functional [48], and the projector augmented-wave (PAW) method [49] was employed to describe the ion–electron interaction. A plane-wave cutoff energy of 700 eV was used throughout all calculations, and the Brillouin zone was sampled with a Monkhorst–Pack grid spacing of 2π×0.03 ${Å}^{-1}$ [50]. All structures were fully relaxed until the total energy and atomic forces converged to within $1\times 10^{-6}$ eV and $1\;\times \;10^{-3}$ eV/Å, respectively. Van der Waals interactions were taken into account using the DFT-D2 correction method [51]. Additional DFT-D3 [52] and DFT-D4 [53] calculations were performed to examine the influence of different dispersion-correction schemes on the adsorption energies, as shown in the Supplementary Materials. Phonon spectra were calculated using the PHONOPY code [54]. To examine thermal stability, ab initio molecular dynamics (AIMD) simulations were carried out in the canonical ensemble using a Nos’e–Hoover thermostat [55,56] at 300 K for 10 ps with a time step of 1 fs.

## 3. Results and Discussion

### 3.1. Crystal Structure and Stability of Li_2_B_2_N_2_ and Li_3_B_2_N_2_ Monolayers

**Figure 1:**
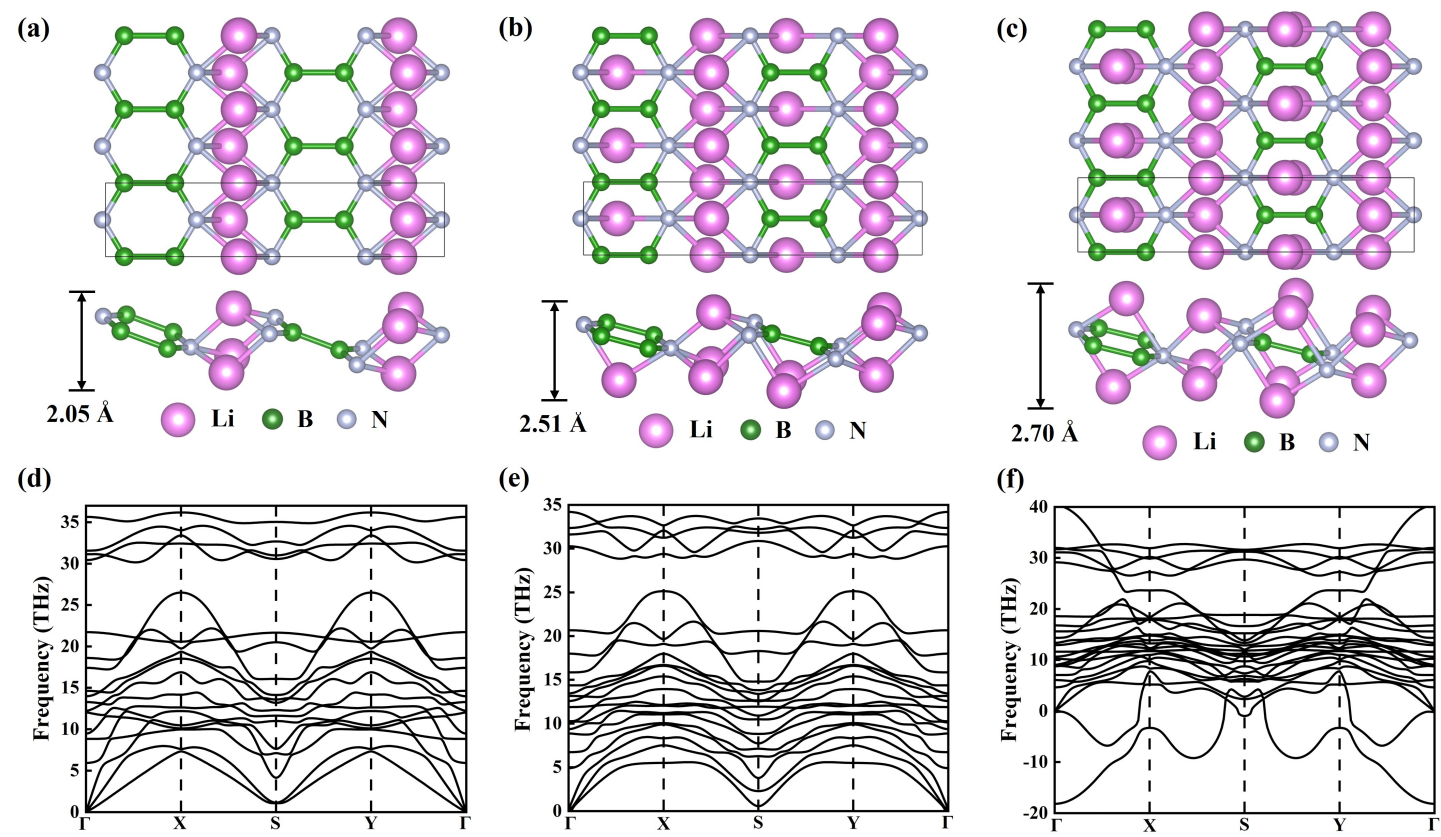
Top and side views of the crystal structures of (**a**) Li_2_B_2_N_2_, (**b**) Li_3_B_2_N_2_, and (**c**) Li_4_B_2_N_2_ monolayers. The pink spheres denote Li atoms at the outer layers, while the green and gray spheres represent B and N atoms in the middle layer, respectively. Phonon dispersion curves of (**d**) Li_2_B_2_N_2_, (**e**) Li_3_B_2_N_2_, and (**f**) Li_4_B_2_N_2_ monolayers.

Figure 1a–c present the optimized crystal structures of Li_2_B_2_N_2_, Li_3_B_2_N_2_, and Li_4_B_2_N_2_ monolayers, as identified from global structure searches. Li_2_B_2_N_2_ and Li_4_B_2_N_2_ crystallize in the *C*2/*m* space group, whereas Li_3_B_2_N_2_ adopts the *Cm* space group. All three monolayers exhibit a similar layered structural motif, in which B and N atoms form a central framework composed of two hexagonal-ring chains, while Li atoms are distributed on the outer layers. In this framework, the B–B bond lengths (1.73–1.75 Å) are notably longer than the B–N bond lengths (1.47–1.50 Å). For Li_2_B_2_N_2_, the Li atoms bridge the two hexagonal-ring chains in an interleaved manner, giving rise to a stable monolayer with a thickness of 2.05 Å. Each Li atom is threefold coordinated by N atoms, with an average Li–N bond length of 2.01 Å. With increasing Li content, the hexagonal B–N framework remains nearly unchanged, while the additional Li atoms occupy the hollow sites located directly above and below the centers of the B_4_N_2_ hexagonal rings. In these positions, each added Li atom is twofold coordinated by N atoms, with an average Li–N bond length of 2.26 Å. As a result, the thickness of the ${\mathrm{Li}}_{n}$B_2_N_2_ (n=2–4) monolayers increases monotonically with Li content, reaching 2.51 Å for Li_3_B_2_N_2_ and 2.70 Å for Li_4_B_2_N_2_. Meanwhile, the progressively increased number of exposed Li sites is expected to be favorable for hydrogen adsorption.

However, excessive Li incorporation reduces the structural stability. As shown in Figure 1d–f, the phonon spectra indicate that Li_2_B_2_N_2_ and Li_3_B_2_N_2_ monolayers are dynamically stable, as no imaginary phonon modes appear throughout the Brillouin zone, whereas Li_4_B_2_N_2_ exhibits pronounced imaginary modes, indicating its dynamical instability. This instability is likely associated with the excessive incorporation of Li atoms. Specifically, the additional Li atoms occupy hollow sites with lower coordination numbers and longer Li–N bond lengths, indicating weaker binding to the B–N framework. Together with the enhanced electrostatic repulsion among Li cations and the increased out-of-plane structural expansion, these factors may soften low-frequency phonon modes and eventually drive the dynamical instability of Li_4_B_2_N_2_.

Next, the thermal stability of Li_2_B_2_N_2_ and Li_3_B_2_N_2_ monolayers was further examined by ab initio molecular dynamics (AIMD) simulations, as shown in Figure S1. It is found that both the total energy and temperature exhibit only small fluctuations throughout the 10 ps simulation at 300 K. Moreover, no obvious atomic displacement or structural degradation is observed. These results confirm that both Li_2_B_2_N_2_ and Li_3_B_2_N_2_ monolayers possess good thermal stability.

To further evaluate the experimental feasibility of Li_2_B_2_N_2_ and Li_3_B_2_N_2_ monolayers, we calculated their cohesive energies ($E_{\mathrm{coh}}$), which are defined as the energy differences between the monolayers and their constituent isolated Li, B, and N atoms. The cohesive energy is expressedas

(1) $$E_{\mathrm{coh}}=\frac{E_{{\mathrm{Li}}_{n}B_{2}N_{2}}-nE_{\mathrm{Li}}-2E_{B}-2E_{N}}{n+4},$$

where $E_{{\mathrm{Li}}_{n}B_{2}N_{2}}$ denotes the total energy of the ${\mathrm{Li}}_{n}$B_2_N_2_ monolayer, $E_{\mathrm{Li}}$, $E_{B}$, and $E_{N}$ are the energies of isolated Li, B, and N atoms, respectively, and *n* is the number of Li atoms. The calculated cohesive energies of Li_2_B_2_N_2_ and Li_3_B_2_N_2_ are −6.22 and −5.59 eV/atom, respectively. These values are comparable to those of experimentally synthesized two-dimensional materials, such as borophene (−5.99 eV/atom) [57], silicene (−4.57 eV/atom) [58], *h*-BN (−7.91 eV/atom) [59], and MoS_2_ (−6.25 eV/atom) [60], indicating their favorable energetic stability and potential experimental feasibility.

### 3.2. Electronic Properties of Li_2_B_2_N_2_ and Li_3_B_2_N_2_ Monolayers

**Figure 2:**
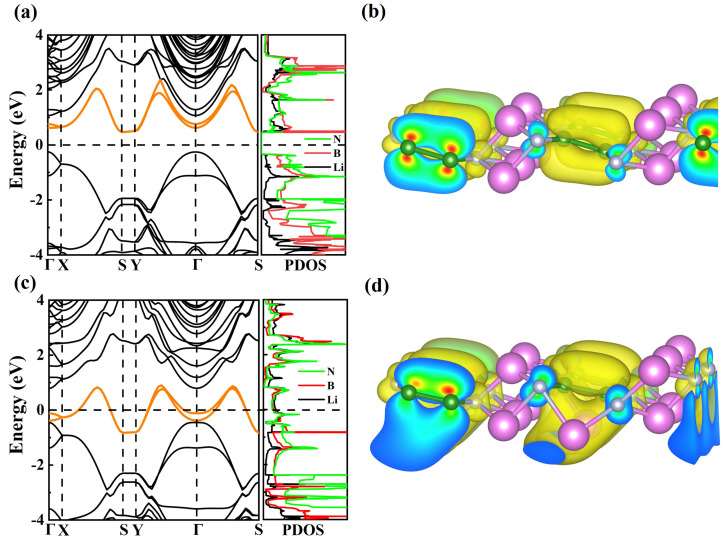
Electronic band structures and the corresponding partial density of states (PDOS) of (**a**) Li_2_B_2_N_2_ and (**c**) Li_3_B_2_N_2_ monolayers. The Fermi level is set to zero. Panels (**b**,**d**) present the band-decomposed charge densities of the orange-highlighted bands near the Fermi level for Li_2_B_2_N_2_ and Li_3_B_2_N_2_, respectively.

The electronic properties of Li_2_B_2_N_2_ and Li_3_B_2_N_2_ monolayers are elucidated by the band structures, partial density of states (PDOS), and band-decomposed charge densities shown in Figure 2. As can be seen, Li_2_B_2_N_2_ is a semiconductor with a small band gap of 0.72 eV, whereas Li_3_B_2_N_2_ exhibits intrinsic metallic behavior, with two bands highlighted in orange crossing the Fermi level ($E_{F}$). The different electronic characters of Li_2_B_2_N_2_ and Li_3_B_2_N_2_ mainly originate from their distinct electron filling. In Li_2_B_2_N_2_, charge transfer from Li to the B–N framework is insufficient to occupy the conduction-band states, leading to semiconducting behavior. In contrast, the additional Li atom in Li_3_B_2_N_2_ provides extra electrons, which partially occupy the B-related $\pi^{*}$ states associated with the B–B bonds [Figure 2b,d]. As a result, the Fermi level is shifted upward into the conduction band, giving rise to intrinsic metallic behavior. The PDOS of both systems shows significant overlapping features among Li, B, and N states, indicating appreciable orbital hybridization and strong interatomic interactions. Given its intrinsic metallicity with a Li-rich surface and stronger local surface electrostatic field, both of which are favorable for polarizing and adsorbing H_2_ molecules, Li_3_B_2_N_2_ is selected for the following investigation of hydrogen storage performance.

### 3.3. Hydrogen Adsorption Behavior of the Li_3_B_2_N_2_ Monolayer

**Figure 3:**
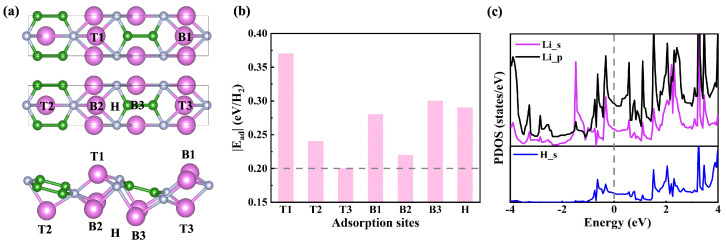
(**a**) Schematic illustration of the possible hydrogen adsorption sites (T, B, and H) on the Li_3_B_2_N_2_ monolayer, corresponding to the top sites above Li atoms, the bridge sites above the midpoints of Li–Li bonds, and the hollow site above the center of the triangle, respectively. (**b**) Calculated hydrogen adsorption energies ($|E_{\mathrm{ad}}|$) for a single H_2_ molecule adsorbed at different sites on the Li_3_B_2_N_2_ monolayer. The gray dashed line marks the lower bound of the optimal adsorption-energy window. (**c**) Projected density of states (PDOS) of the Li_3_B_2_N_2_ monolayer with one H_2_ molecule adsorbed at the B3 site. The Fermi level ($E_{F}$) is set to zero.

We next examine the adsorption behavior of a single H_2_ molecule on the Li_3_B_2_N_2_ monolayer. As shown in Figure 3a, seven possible adsorption sites are considered, including three top sites above Li atoms (T1, T2, and T3), three bridge sites above the midpoints of Li–Li bonds (B1, B2, and B3), and one hollow site above the center of the triangle (H). These sites cover the main high-symmetry adsorption positions on the monolayer surface. The optimized structures of H_2_@Li_3_B_2_N_2_ are shown in Figure S2.

The hydrogen adsorption energy, $E_{\mathrm{ad}}$, is calculated as

(2) $$E_{\mathrm{ad}}=\frac{E_{nH_{2}@{\mathrm{Li}}_{3}B_{2}N_{2}}-E_{{\mathrm{Li}}_{3}B_{2}N_{2}}-nE_{H_{2}}}{n},$$

where $E_{nH_{2}@{\mathrm{Li}}_{3}B_{2}N_{2}}$, $E_{{\mathrm{Li}}_{3}B_{2}N_{2}}$, and $E_{H_{2}}$ denote the total energies of *n*H_2_@Li_3_B_2_N_2_ system, pristine Li_3_B_2_N_2_ monolayer, and an isolated H_2_ molecule, respectively, and *n* is the number of adsorbed H_2_ molecules.

The calculated absolute value of adsorption energies $|E_{\mathrm{ad}}|$ for a single H_2_ molecule at these sites are summarized in Figure 3b. All adsorption configurations are energetically favorable, with adsorption energies $|E_{\mathrm{ad}}|$ ranging from 0.20 to 0.37 eV/H_2_. Among them, the T1 site gives the highest adsorption energy of 0.37 eV/H_2_, whereas the T3 site shows the weakest adsorption, with an adsorption energy of 0.20 eV/H_2_. The B1, B3, and H sites exhibit adsorption energies of 0.28, 0.30, and 0.29 eV/H_2_, respectively, while the T2 and B2 sites give slightly smaller values of 0.24 and 0.22 eV/H_2_. Notably, all these values fall within or very close to the desirable adsorption-energy window of 0.2–0.6 eV/H_2_ for reversible hydrogen storage under ambient conditions, as suggested by the U.S. Department of Energy (DOE) [6]. Although the T1 site shows a slightly higher adsorption energy, adsorption at this site induces noticeable structural distortion (Figure S2). Therefore, the B3 site is identified as the most favorable adsorption configuration, with an adsorption energy of 0.30 eV/H_2_ without inducing obvious structural distortion, which is favorable for reversible hydrogen storage under near-ambient conditions.

To further understand the adsorption mechanism, we analyze the projected density of states (PDOS) of the B3 adsorption configuration, as shown in Figure 3c. Clear overlap between the H-*s* state and the Li-s,p states can be observed in several energy regions, indicating noticeable interaction between the adsorbed H_2_ molecule and the Li sites. This result suggests that H_2_ adsorption on Li_3_B_2_N_2_ is not governed solely by van der Waals interaction, but also involves weak orbital hybridization. The positively charged Li atoms on the surface create a strong local electrostatic field, which can polarize the H_2_ molecule. At the same time, weak orbital hybridization also contributes to the interaction. Therefore, the adsorption of H_2_ arises from the combined effects of electrostatic polarization and orbital hybridization.

### 3.4. Hydrogen Storage Capacity of the Li_3_B_2_N_2_ Monolayer

To further evaluate the hydrogen storage capacity of the Li_3_B_2_N_2_ monolayer, we gradually increased the number of adsorbed H_2_ molecules on the 1×3×1 supercell. A 1×3×1 supercell was adopted in the subsequent calculations, because the supercell-size convergence test shows that the adsorption energy is already well converged at this size. As shown in Figure S3, the adsorption energy remains nearly unchanged when the supercell is further enlarged from 1×3×1 to 1×5×1, indicating that the interaction between periodically repeated H_2_ molecules is negligible. Therefore, the 1×3×1 supercell provides a reliable and computationally efficient model.

**Figure 4:**
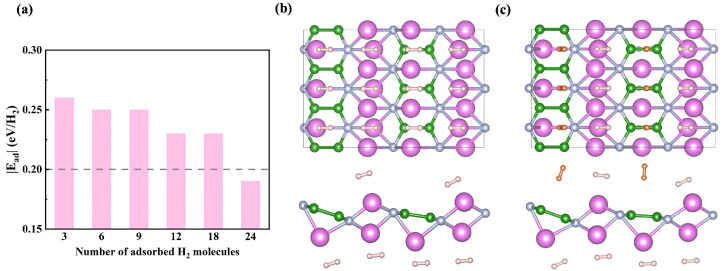
(**a**) Average adsorption energies ($|E_{\mathrm{ad}}|$) of H_2_ molecules as a function of the number of adsorbed H_2_ molecules on the 1×3×1 supercell of the Li_3_B_2_N_2_ monolayer. The gray dashed line marks the lower bound of the optimal adsorption-energy window. Optimized structures of (**b**) 18H_2_@Li_18_B_12_N_12_ and (**c**) 24H_2_@Li_18_B_12_N_12_. The desorbed H_2_ molecules are highlighted in orange.

As shown in Figure 4a, the average adsorption energy $|E_{\mathrm{ad}}|$ remains within the favorable range when the number of adsorbed H_2_ molecules increases from 3 to 18, with values of 0.23–0.26 eV/H_2_. This indicates that the Li_3_B_2_N_2_ monolayer can maintain moderate and reversible interactions with multiple H_2_ molecules. When the number of H_2_ molecules further increases to 24, the average adsorption energy decreases to 0.19 eV/H_2_, slightly below the lower bound of the desirable adsorption-energy window. The optimized structures in Figure 4b,c further show that all 18 H_2_ molecules remain stably adsorbed, whereas six H_2_ molecules (highlighted in orange) desorb from the surface in the 24 H_2_ configuration. This behavior can be attributed to the steric hindrance and intermolecular repulsion between adjacent H_2_ molecules at high coverage. The optimized structures of the remaining H_2_ adsorption configurations are shown in Figure S4. These results indicate that the maximum stable adsorption capacity of the Li_3_B_2_N_2_ monolayer is 18 H_2_ molecules per 1×3×1 supercell.

In 18H_2_@Li_18_B_12_N_12_ structure, the adsorbed H_2_ molecules retain H–H bond lengths of 0.757–0.812 Å, close to the isolated-H_2_ value of 0.750 Å. Bader charge analysis shows that each adsorbed H_2_ molecule obtained 0.155 e, consistent with the charge density difference map shown in Figure S5a, suggesting a weak interaction between H_2_ molecule and substrate. The reduced density gradient (RDG) analysis [shown in Figure S5b] further confirm the weak interaction (electrostatic polarization and weak van der Waals interactions) dominated molecular adsorption of H_2_.

The hydrogen storage capacity was calculated as

(3) $$G_{C}\;\left(\mathrm{wt}.\%\right)=\frac{n_{H_{2}}w_{H_{2}}}{n_{\mathrm{Li}}w_{\mathrm{Li}}+n_{B}w_{B}+n_{N}w_{N}+n_{H_{2}}w_{H_{2}}}\times 100\%,$$

where $n_{H_{2}}$, $n_{\mathrm{Li}}$, $n_{B}$, and $n_{N}$ are the numbers of H_2_ molecules, Li, B, and N atoms, respectively, while $w_{H_{2}}$, $w_{\mathrm{Li}}$, $w_{B}$, and $w_{N}$ denote the corresponding molecular or atomic weights. Therefore, 18 H_2_ molecules adsorbed on the 1×3×1 supercell represent the maximum stable adsorption capacity of the Li_3_B_2_N_2_ monolayer, corresponding to a gravimetric hydrogen storage capacity of 7.8 wt.%.

As summarized in Table 1, we compare the adsorption energy and gravimetric hydrogen storage capacity of Li_3_B_2_N_2_ with those of representative two-dimensional hydrogen storage materials. Pristine 2D materials, such as graphene and h-BN, generally exhibit weak H_2_ adsorption energy and limited storage capacity, while metal-decorated systems, such as Li@Irida-graphene, Li@BC_2_N, and Li@AsC_5_, show improved hydrogen-storage performance. Li_3_B_2_N_2_ exhibits a competitive hydrogen storage capacity of 7.8 wt.% with a moderate adsorption energy of 0.23 eV/H_2_, comparable to these recently reported metal-decorated 2D materials. Notably, this performance on Li_3_B_2_N_2_ is achieved by intrinsic Li-rich adsorption sites without external metal decoration, underscoring Li_3_B_2_N_2_ as a promising metal-decoration-free 2D hydrogen storage material.

### 3.5. Desorption Capacity of the H_2_ Molecule on the Li_3_B_2_N_2_ Monolayer

Beyond achieving a high hydrogen storage capacity, an onboard hydrogen storage system must also remain stable under practical operating conditions, namely, it should be able to reversibly adsorb and release hydrogen at room temperature and moderate pressures (0.2∼12 MPa). Therefore, the effects of temperature and pressure on the structural stability were evaluated based on the relative energy $E_{r}$, defined as

(4) $$E_{r}=E_{nH_{2}@{\mathrm{Li}}_{3}B_{2}N_{2}}-E_{{\mathrm{Li}}_{3}B_{2}N_{2}}-n\left[E_{H_{2}}+\mu_{H_{2}}\left(T,P\right)\right],$$

where $E_{nH_{2}@{\mathrm{Li}}_{3}B_{2}N_{2}}$, $E_{{\mathrm{Li}}_{3}B_{2}N_{2}}$, $E_{H_{2}}$, and *n* have the same meanings as those in Equation (2). $\mu_{H_{2}}\left(T,P\right)$ is the chemical potential of hydrogen at temperature *T* and pressure *P*, which can be expressed as

(5) $$\mu_{H_{2}}\left(T,P\right)=\Delta H-T\Delta S+k_{B}T\ln\left(\frac{P}{P^{0}}\right),$$

where $P^{0}$ is the standard pressure of 0.1 MPa, $k_{B}$ is the Boltzmann constant, and ΔH(T) and ΔS(T) are the enthalpy and entropy changes of gas-phase H_2_ from 0 K to the target temperature at ambient pressure. The values of ΔH−TΔS were taken from the thermochemical tables [63] and are listed in Table 2.

**Figure 5:**
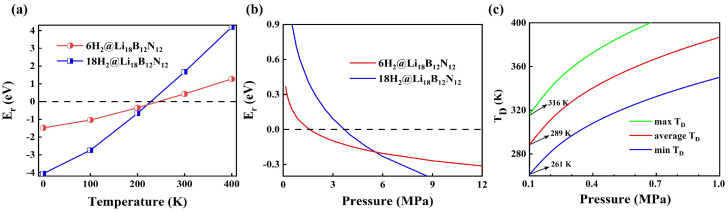
(**a**) Relative energies ($E_{r}$) of 6H_2_@Li_18_B_12_N_12_ and 18H_2_@Li_18_B_12_N_12_ as functions of temperature under standard atmospheric pressure (p=0.1 MPa). (**b**) Relative energies ($E_{r}$) of 6H_2_@Li_18_B_12_N_12_ and 18H_2_@Li_18_B_12_N_12_ as functions of pressure at 300 K. (**c**) Desorption temperature ($T_{D}$) as a function of atmosphere pressure. The green, red, and blue curves represent the maximum, average, and minimum $T_{D}$ values, respectively.

Figure 5a shows the relative energies ($E_{r}$) of 6H_2_@Li_18_B_12_N_12_ and 18H_2_@Li_18_B_12_N_12_ as a function of temperature at standard atmospheric pressure. At 0 K, both 6H_2_@Li_18_B_12_N_12_ and 18H_2_@Li_18_B_12_N_12_ have negative relative energies, indicating that these adsorption structures are thermodynamically stable. As the temperature increases, the relative energies gradually increase and reach zero at about 245 K for 6H_2_@Li_18_B_12_N_12_ and 229 K for 18H_2_@Li_18_B_12_N_12_. Above these temperatures, the adsorbed H_2_ molecules become thermodynamically unfavorable and tend to desorb spontaneously. Figure 5b presents the relative energies of 6H_2_@Li_18_B_12_N_12_ and 18H_2_@Li_18_B_12_N_12_ as a function of pressure at 300 K. The minimum equilibrium pressures are estimated to be about 1.6 MPa for 6H_2_@Li_18_B_12_N_12_ and 3.7 MPa for 18H_2_@Li_18_B_12_N_12_. These results indicate that the Li_3_B_2_N_2_ monolayer can achieve reversible hydrogen storage through external pressure control, that is, hydrogen adsorption can be realized at pressures above 3.7 MPa.

The desorption temperature ($T_{D}$) of the Li_3_B_2_N_2_-based hydrogen storage system was further estimated using the van’t Hoff equation:

(6) $$T_{D}=\frac{E_{\mathrm{ad}}}{k_{B}{\left[\left(\Delta S/R\right)-\ln p\right]}^{-1}}.$$

Here, $E_{\mathrm{ad}}$ is the hydrogen adsorption energy, $k_{B}$ is the Boltzmann constant, ΔS is the entropy change from gaseous hydrogen to adsorbed hydrogen, which is approximately equal to the standard entropy of hydrogen gas, 130 J/(K·mol), *R* is the gas constant (R=8.31 J/(K·mol)), and *p* is the equilibrium pressure.

Here, the minimum and maximum desorption temperatures are approximately estimated from representative adsorption energies at different H_2_ coverages. The 24 H_2_ configuration, with relatively weaker adsorption at high coverage, is taken to describe the onset of H_2_ desorption, whereas the 9 H_2_ configuration, with stronger adsorption at lower coverage, is used to represent the final stage of hydrogen release. The 18 H_2_ configuration, corresponding to the maximum stable loading, is adopted to evaluate the average desorption temperature. The pressure-dependent desorption-temperature curves at the minimum, average, and maximum $T_{D}$ values are shown in Figure 5c. At standard atmospheric pressure (∼0.1 MPa), the maximum, average, and minimum desorption temperatures are 316, 289, and 261 K, respectively. Increasing the pressure further raises the desorption temperature. Furthermore, the H_2_ occupation analysis for 18H_2_@Li_18_B_12_N_12_ based on a simplified grand-canonical thermodynamic model [25,26,64,65], presented in the Supplementary Materials, shows a consistent temperature- and pressure-dependent trend. Therefore, these thermodynamic results show that H_2_ adsorption and desorption on Li_3_B_2_N_2_ can be reversibly controlled by temperature and pressure. The predicted desorption near-room-temperature range of 261–316 K at 0.1 MPa is highly favorable for fuel-cell applications, highlighting Li_3_B_2_N_2_ as a promising candidate for reversible hydrogen storage under near-ambient conditions.

## 4. Conclusions

In summary, based on crystal structure prediction and first-principles calculations, we have identified Li_3_B_2_N_2_ as a promising intrinsically Li-rich two-dimensional material for reversible hydrogen storage. Structural analysis shows that Li_2_B_2_N_2_ and Li_3_B_2_N_2_ monolayers are both dynamically and thermally stable, whereas excessive Li incorporation leads to the dynamical instability of Li_4_B_2_N_2_. Electronic structure calculations reveal that Li_3_B_2_N_2_ exhibits intrinsic metallic behavior and an enhanced local surface electrostatic field, both of which are favorable for hydrogen adsorption. The adsorption energies of a single H_2_ molecule on Li_3_B_2_N_2_ range from 0.20 to 0.37 eV/H_2_, which fall within the desirable window for reversible hydrogen storage. The adsorption mechanism is mainly governed by the combined effects of electrostatic polarization and weak orbital hybridization. Further calculations show that the Li_3_B_2_N_2_ monolayer can accommodate up to 18 H_2_ molecules in a 1×3×1 supercell, corresponding to a hydrogen storage capacity of 7.8 wt.%. Furthermore, thermodynamic analysis demonstrates that H_2_ adsorption and desorption can be regulated by temperature and pressure, and the predicted desorption temperature range of 261–316 K at 0.1 MPa suggests favorable reversibility under near-ambient conditions. These results demonstrate that Li_3_B_2_N_2_ is a highly promising intrinsic 2D material for high-capacity and reversible hydrogen storage, and provide a useful design strategy for developing next-generation metal-decoration-free hydrogen storage materials.

## Figures and Tables

**Table 1:** Summary of the hydrogen adsorption Energies ($\left|E_{\mathrm{ad}}\right|$), hydrogen storage capacities, and desorption temperature ($T_{D}$) of common 2D materials and Li decorated 2D materials.

Compound	$\left|E_{\mathrm{ad}}\right|$ [eV/H_2_]	Storage Capacity [wt.%]	$T_{D}$	Refs.
Li_3_B_2_N_2_	0.23	7.8	261–316 K/0.1 MPa	This work
graphene	…	0.4	77 K/100 kPa	[8]
*h*-BN	…	2.96	243 K/10 MPa	[9]
Li@graphene	0.56	12.8	…	[22]
Li@Irida-graphene	0.23–0.28	7.06	353 K/0.1 MPa	[23]
Li-doped γ-graphdiyne	0.21–0.23	14.66	…	[24]
Li@T-BN	0.25–0.32	12.31	180–232.6 K/1 atm	[28]
Li@B_2_N	0.19–0.27	11.1	<200 K/0.1 MPa	[30]
Li@penta-BCN	0.16	7.44	…	[31]
Li@BC_2_N	0.18–0.30	11.10	360 K/1 atm	[32]
Li@POG-B_4_C_2_N_3_	0.19–0.35	8.35	…	[33]
Li@AsC_5_	∼0.19	9.7	243–357 K/1 atm	[61]
Li@GeC_5_	0.22	7.62	281.1 K/1 atm	[62]

**Table 2:** Values of ΔH−TΔS obtained from the thermochemical tables.

*T* [K]	ΔS(T) [J/(mol · K)]	ΔH(T) [kJ/mol]	ΔH(T)−TΔS(T) [eV/H_2_]
0	0	0	0
100	100.73	3.00	−0.073
200	119.41	5.69	−0.189
300	130.86	8.52	−0.319
400	139.216	11.43	−0.459

## Data Availability

The original contributions presented in this study are included in the article/Supplementary Materials. Further inquiries can be directed to the corresponding author.

## Supplementary Materials

The following supporting information can be downloaded at: https://www.mdpi.com/article/10.3390/nano16110654/s1, Figure S1: Energy fluctuations during MD simulations at 300 K of (a) Li_2_B_2_N_2_ and (b) Li_3_B_2_N_2_ monolayers. The insets in (a) and (b) display the side and top view of the structural snapshots at the end of the 10 ps simulations; Figure S2: Optimized structures of H_2_ adsorbed at different adsorption sites on the Li_3_B_2_N_2_ monolayer, shown in top and side views. The deep pink, green, gray, and light pink spheres denote Li, B, N, and H atoms, respectively; Figure S3: (a) Convergence of the hydrogen adsorption energy ($\left|E_{\mathrm{ad}}\right|$) with respect to the supercell size for Li_3_B_2_N_2_ with one adsorbed H_2_ molecule. (b) Top and side views of the optimized 1 × 3 × 1 Li_3_B_2_N_2_ supercell with one H_2_ molecule adsorbed at the B3 site; Figure S4: Optimized structures of (a) three, (b) six, (c) nine, and (d) twelve H_2_ molecules absorbed on the 1 × 3 × 1 supercell of the Li_3_B_2_N_2_ monolayer; Figure S5: (a) Charge density difference map with an isosurface value of $0.0016\;e/{Å}^{3}$ and (b) reduced density gradient (RDG) analyses of the 18H_2_@Li_18_B_12_N_12_ adsorption configuration; Table S1: The optimized structural parameters of the Li_3_B_2_N_2_ and Li_2_B_2_N_2_; Table S2: Comparison of H_2_ adsorption energies $E_{\mathrm{ad}}$ calculated using different van der Waals correction schemes; Table S3: The H_2_ occupation θ of 18H_2_@Li_18_B_12_N_12_ as a function of temperature under standard atmospheric pressure (P = 0.1 MPa); Table S4: The H_2_ occupation θ of 18H_2_@Li_18_B_12_N_12_ as a function of pressure at 300 K.
