# Supplementary material for: High-Capacityand Reversible Hydrogen Storage in an Intrinsic Li3B2N2 Monolayer
Source: Nanomaterials (Basel). 2026 May 23;16(11):654. doi: 10.3390/nano16110654 (PMC13257736; doi:10.3390/nano16110654)
Supplement: Supplementary file 1 [file nanomaterials-16-00654-s001.zip › nanomaterials-4305654-supplementary.pdf]

nanomaterials

Supplementary information for "High-Capacity and Reversible Hydrogen Storage in an Intrinsic $Li_3B_2N_2$ Monolayer"

Haichuan Yu[a], Jingyan Chen[a], Jian Hao[*a], Caoping Niu[*a], Meiling Xu[*a], and Yinwei Li[a]

[a] *Jiangsu Key Laboratory of Extreme Multi-Field Materials Physics, School of Physics and Electronic Engineering, Jiangsu Normal University, Xuzhou 221116, China*

Author to whom all correspondence should be addressed: jian_hao@jsnu.edu.cn, cpniu@jsnu.edu.cn, xml@calypso.cn

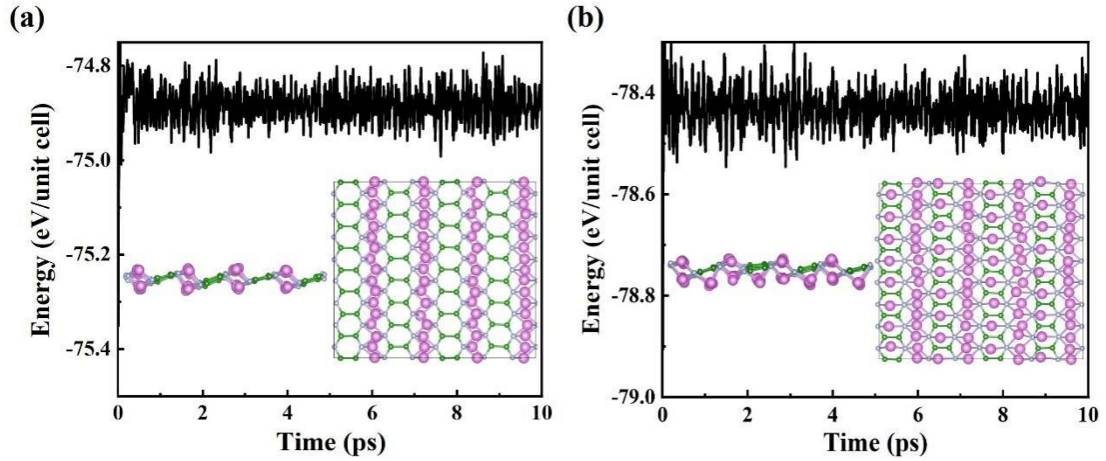

Figure S1. Energy fluctuations during MD simulations at 300 K of (a) $Li_2B_2N_2$ and (b) $Li_3B_2N_2$ monolayers. The insets in (a) and (b) display the side and top view of the structural snapshots at the end of the 10 ps simulations.

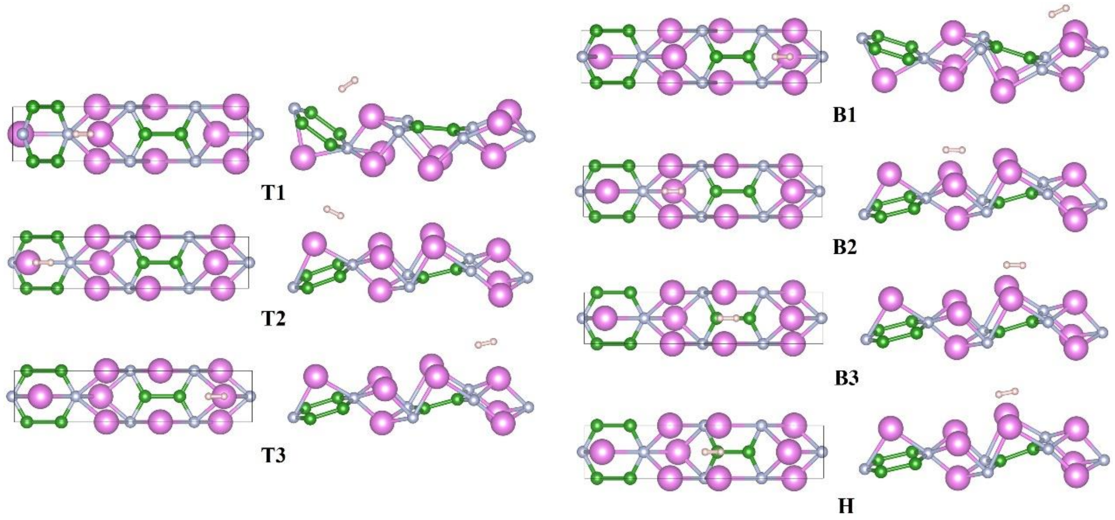

Figure S2. Optimized structures of H_2 adsorbed at different adsorption sites on the $Li_3B_2N_2$ monolayer, shown in top and side views. The deep pink, green, gray, and light pink spheres denote Li, B, N, and H atoms, respectively.

https://doi.org/10.3390/nano16110654

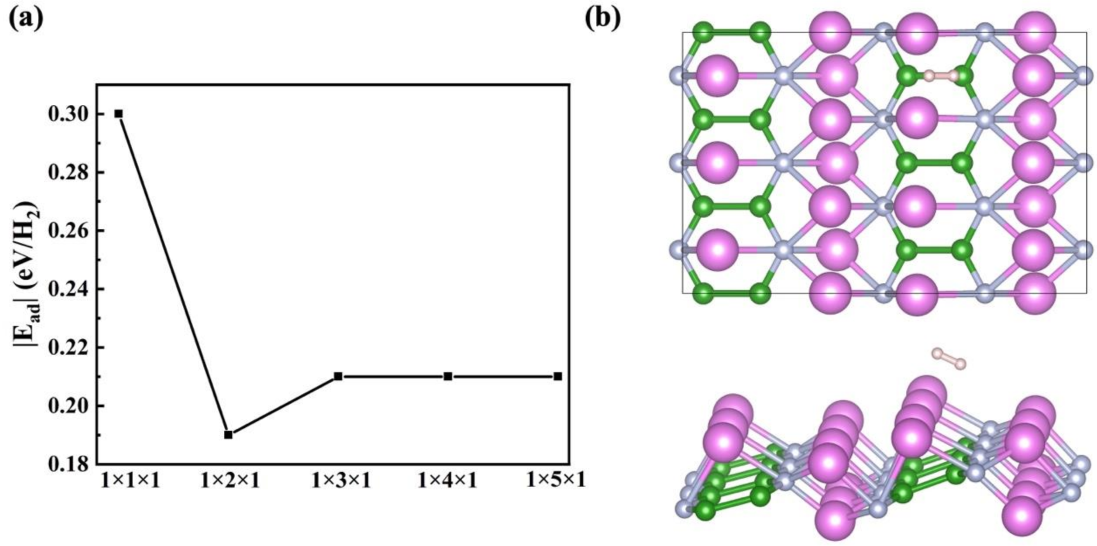

Figure S3. (a) Convergence of the hydrogen adsorption energy ($|E_{ad}|$) with respect to the supercell size for $Li_3B_2N_2$ with one adsorbed H_2 molecule. (b) Top and side views of the optimized 1×3×1 $Li_3B_2N_2$ supercell with one H_2 molecule adsorbed at the B3 site.

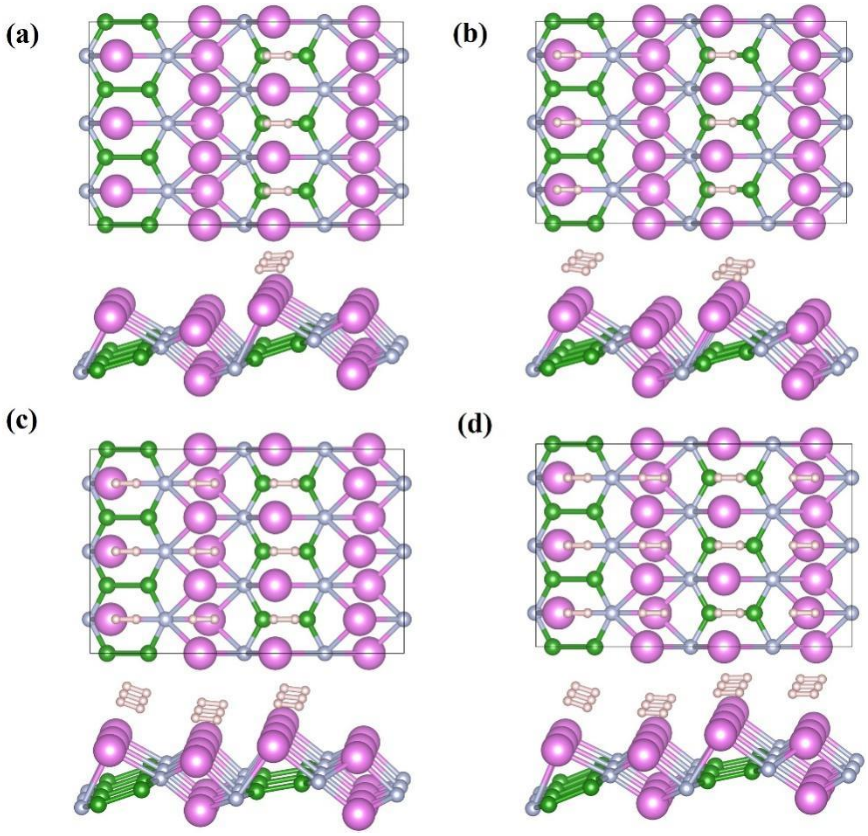

Figure S4. Optimized structures of (a) three, (b) six, (c) nine, and (d) twelve H_2 molecules absorbed on the 1×3×1 supercell of the $Li_3B_2N_2$ monolayer.

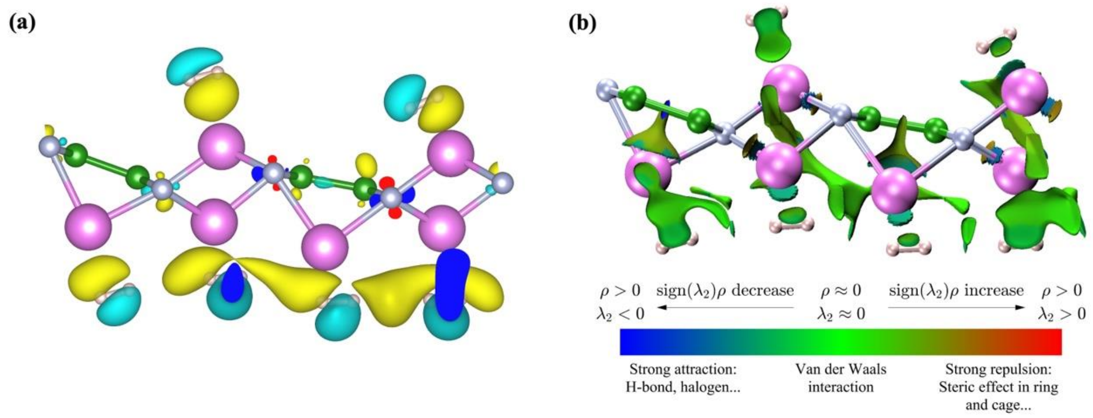

Figure S5. (a) Charge density difference map with an isosurface value of 0.0016 e/Å^3 and (b) reduced density gradient (RDG) analyses of the $18H_2@Li_{18}B_{12}N_{12}$ adsorption configuration.

Table S1. The optimized structural parameters of the $Li_2B_2N_2$ and $Li_3B_2N_2$ monolayers.

Compounds	Space group	a, b, c (Å)<br>α, β, γ (deg)	Atomic position
**$Li_2B_2N_2$**	***C*2/*m***	a=11.88540<br>b=2.53130<br>c=29.63570<br>α=γ=90<br>β=101.4744	Li1(4i) (0.23615, 0.50000, 0.46534)<br>B1(4i) (0.42553, 0.50000, 0.49085)<br>N1(4i) (0.63892, 0.00000, 0.51614)
**$Li_3B_2N_2$**	***Cm***	a=12.0199<br>b=2.57680<br>c=24.0199<br>α=γ=90<br>β=89.5512	Li1(2a) (0.09937, 0.50000, 0.48191)<br>Li2(2a) (0.36549, 0.00000, 0.49949)<br>Li3(2a) (0.87749, 0.00000, 0.58631)<br>B1(2a) (0.19259, 0.00000, 0.53991)<br>B2(2a) (0.05204, 0.00000, 0.55657)<br>N1(2a) (0.99206, 0.50000, 0.56319)<br>N2(2a) (0.25162, 0.50000,0.53136)

https://doi.org/10.3390/nano16110654

Table S2. Comparison of H_2 adsorption energies E_{ad} calculated using different van der Waals correction schemes.

**Configure**	**E_{ad} with DFT-D2 [eV/H_2]**	**E_{ad} with DFT-D3 [eV/H_2]**	**E_{ad} with DFT-D4 [eV/H_2]**	**Discrepancy**
**T1**	-0.369	-0.346	-0.304	0.065
**T2**	-0.242	-0.237	-0.227	0.015
**T3**	-0.196	-0.221	-0.211	0.025
**B1**	-0.283	-0.296	-0.251	0.046
**B2**	-0.217	-0.182	-0.175	0.042
**B3**	-0.299	-0.228	-0.223	0.075
**H**	-0.290	-0.225	-0.304	0.079
**$18H_2@Li_{18}B_{12}N_{12}$**	-0.227	-0.127	-0.114	0.114

https://doi.org/10.3390/nano16110654

Table S3. The H_2 occupation θ of $18H_2@Li_{18}B_{12}N_{12}$ as a function of temperature under standard atmospheric pressure (P = 0.1 MPa).

**T [K]**	μ_{H_2} **[eV/H2]**	G_{ad} **[eV/H2]**	θ	$\langle n \rangle$
**0**	0	-0.227	1	18
**100**	-0.073	-0.154	1	18
**200**	-0.189	-0.038	0.902	16.24
**300**	-0.319	0.092	0.028	0.5
**400**	-0.459	0.232	0.0012	0.02

TABLE S4. The H_2 occupation θ of $18H_2@Li_{18}B_{12}N_{12}$ as a function of pressure at 300 K.

**P [MPa]**	μ_{H_2} **[eV/H2]**	G_{ad} **[eV/H2]**	θ	$\langle n \rangle$
**0**	-∞	+∞	0	0
**3**	-0.231	0.0038	0.463	8.34
**6**	-0.213	-0.014	0.633	11.40
**9**	-0.203	-0.025	0.721	12.99
**12**	-0.195	-0.032	0.775	13.96

We have added a simplified grand-canonical thermodynamic model to estimate the H_2 occupation $\theta(T,P)$ on $Li_3B_2N_2$ as a function of temperature and pressure, as described by the following equations:

The adsorption free energy $G_{ad}(T,P)$ per H_2 molecule was evaluated as

$$G_{ad}(T,P) = E_{ad} - \mu_{H_2}(T,P)$$

The H_2 occupation probability $\theta(T,P)$was estimated using a Langmuir-type expression,

$$\theta(T,P) = \frac{1}{1 + e^{\frac{G_{ad}(T,P)}{k_B T}}}$$

https://doi.org/10.3390/nano16110654

where $\theta(T,P)$ is the fractional occupation of the available H_2 adsorption sites. k_B is the Boltzmann constant. The average number of adsorbed H_2 molecules $\langle n \rangle$ was obtained as

$$\langle n \rangle = n_{max}\,\theta(T,P)$$

where n_{max} is the maximum number of stably adsorbed H_2 molecules. In this work, $n_{max} = 18$ for the 1 × 3 × 1 $Li_{18}B_{12}N_{12}$ supercell.

This model provides an approximate thermodynamic estimate of the H_2 occupation as a function of temperature and pressure, based on the average adsorption free energy evaluated from the DFT adsorption energy and the chemical-potential correction of gas-phase H_2 [1]. Similar occupation analyses have been applied to Li-doped biphenylene [2], Sc-decorated two-dimensional polyaramid systems [3], and Ti/Zr-decorated two-dimensional polyaramid systems [4] to evaluate their hydrogen storage performance under practical operating conditions.

The occupation analysis shows that the H_2 occupation gradually decreases as the temperature increases, and $18H_2@Li_{18}B_{12}N_{12}$ retains a high occupation at 200 K under 0.1 MPa. At 300 K, the H_2 occupation increases with pressure, reaching approximately 14 adsorbed H_2 molecules at 12 MPa. This temperature–pressure dependence indicates that hydrogen adsorption and desorption on $Li_{18}B_{12}N_{12}$ are thermodynamically tunable, supporting reversible H_2 storage under near-room-temperature and moderate-pressure conditions.

Reference:

1. Lee, H.; Choi, W.I.; Ihm, J. Combinatorial Search for Optimal Hydrogen-Storage Nanomaterials Based on Polymers. Phys. Rev. Lett. 2006, 97, 056104.
2. Beniwal, P.; Sagar, S.; Dange, D.; Dhilip Kumar, T.J. Li Doping in Two-Dimensional Holey Biphenylene Framework for Efficient Molecular Hydrogen Storage: A First-Principles Insight. Energy & Fuels 2025, 39, 22083–22094.
3. Vaidyanathan, A.; Wagh, V.; Chakraborty, B. Enhancing Hydrogen Storage Efficiency: Computational Insights into Scandium-decorated 2D Polyaramid. Journal of Power Sources 2024, 624, 235546.
4. Vaidyanathan, A.; Mane, P.; Wagh, V.; Chakraborty, B. Computational Design for Enhanced Hydrogen Storage on the Newly Synthesized 2D Polyaramid via Titanium and Zirconium Decoration. ACS Applied Materials & Interfaces 2024, 16, 8589–8602.

Disclaimer/Publisher's Note: The statements, opinions and data contained in all publications are solely those of the individual author(s) and contributor(s) and not of MDPI and/or the editor(s). MDPI and/or the editor(s) disclaim responsibility for any injury to people or property resulting from any ideas, methods, instructions or products referred to in the content.

https://doi.org/10.3390/nano16110654
